# Taxonomy, tissue, and habitat influence mollusk microbial communities

**DOI:** 10.1093/ismejo/wrag092

**Published:** 2026-04-22

**Authors:** Diana Carolina Vergara-Florez, Thomas F Duda

**Affiliations:** Department of Ecology & Evolutionary Biology, University of Michigan, 1105 North University Avenue, Biological Sciences Building, Ann Arbor, MI 48109-1085, United States; Department of Ecology & Evolutionary Biology, University of Michigan, 1105 North University Avenue, Biological Sciences Building, Ann Arbor, MI 48109-1085, United States

**Keywords:** host−microbiome interactions, microbial community assembly, phylosymbiosis, Mollusca, host phylogeny, marine invertebrates, intrinsic and extrinsic factors, microbiome diversity, predictive modeling

## Abstract

Microbes play a crucial role in the health, development, and resilience of mollusks, yet the ecological and evolutionary factors shaping their microbial communities remain poorly understood. To uncover the drivers of microbial community composition of mollusks, we conducted a systematic review of 85 studies, including 45 on bivalves from marine and freshwater habitats; 33 on gastropods from marine, freshwater, and terrestrial habitats; and seven on cephalopods. Our synthesis reveals that both intrinsic (e.g. host phylogeny, tissue type) and extrinsic (e.g. environment, geography, and seasonality) factors influence microbial community structure, but the effects are highly taxon- and context-dependent. Although studies of bivalves often emphasize environmental drivers, those of cephalopods more frequently highlight intrinsic host features. Despite growing interest in molluscan microbiomes, we identified significant taxonomic and methodological biases, including a predominant focus on economically important species and gut tissues. We advocate for a broader, integrative approach that includes underrepresented molluscan groups, diverse tissue types, and testing of both intrinsic and extrinsic variables across spatial and temporal gradients. This review highlights the need for standardized, multi-factorial research to better understand and predict microbial community responses to environmental change of one of the most diverse and ecologically important invertebrate phyla.

## Introduction

Host-associated microbial communities, or microbiomes, are essential components of nearly all ecosystems, playing crucial roles in host health, development, immunity, and resilience across the tree of life [1–4]. From an ecological and evolutionary perspective, microbiome structure emerges from the interplay of four fundamental processes: selection, dispersal, drift, and diversification [5]. Selection reflects deterministic pressures that favor specific microbial taxa, such as host immune filtering, tissue biochemistry, or environmental conditions. Dispersal determines which microbes reach a given host or habitat, shaping colonization opportunities across space and time. Drift introduces stochastic fluctuations in community composition, particularly in small or isolated populations. At the same time, diversification creates microbial lineages that may adapt to host-specific niches. Together, these processes act simultaneously and interactively, generating the complex patterns of microbiome assembly observed across animal systems [6, 7]. Beyond these classical ecological processes, often signaling host filtering or environmental selection, microbiome structure can emerge from stochastic dispersal and ecological drift alone (i.e. the Sloan neutral model, which predicts the abundance of taxa under purely neutral dynamics) [8, 9]. These neutral models provide an important complementary framework for interpreting microbial community assembly to quantify the relative contributions of stochasticity. Establishing this conceptual foundation is essential for determining how different factors drive microbial community variation in mollusks and other animal taxa.

In animals, the structure of these communities (i.e. the composition, diversity, and abundance of microbes) is shaped by a complex interplay between intrinsic (host-related) and extrinsic (environmental) factors that govern which microorganisms colonize, persist, or are excluded. Understanding the factors that structure these communities is central to interpreting host–microbe interactions, reconstructing evolutionary processes, and predicting how microbiomes may respond to rapidly changing environments.

Intrinsic drivers include phylogenetic relationships of the hosts (host phylogeny—interspecific), genetic traits (intraspecific), tissue type, life stage, sex, and mode of reproduction, all of which can influence host–microbe compatibility, immune responses, and niche availability [1, 10, 11]. Among these, phylogenetic relationships of hosts are one of the most consistent predictors of microbial composition with phylosymbiosis [12–14], the mirroring of host evolutionary history by microbiota, observed across mammals, birds, insects, and some terrestrial [15, 16] and marine invertebrates [17] such as corals [18, 19] and sponges [20]. Other intrinsic traits, such as reproductive mode (sexual vs. asexual), sex, and developmental stage, influence microbiome structure via physiological, hormonal, and immune differences, often reinforced by vertical microbial transmission [21–23]. Tissue type also plays a key role: microbial assemblages can differ between internal and external host surfaces due to variation in function and exposure.

Extrinsic factors, in contrast, encompass environmental variables such as geography, microhabitat (i.e. collection site), temperature, salinity, humidity, seasonality, diet, and anthropogenic pressures (e.g. captivity associated with agriculture and aquaculture practices). Geographic location often correlates with local microbial pools and abiotic gradients, whereas captivity has been linked repeatedly to reduced microbial diversity and functional shifts, highlighting the complex interplay between host biology and environmental context in shaping microbial community structure [24–26].

In a range of invertebrate taxa, host genetics and environmental context play competing roles in shaping microbiome composition. Marine invertebrates such as corals and sponges exhibit the dual influence of intrinsic and extrinsic factors on microbiome composition [27–30]. For example, host phylogeny, depth, and ocean basin are often the primary drivers of microbial structure of corals, with additional variation attributed to microhabitat conditions and sampling time [31]. In addition, sponges exhibit similarly complex patterns: microbial community composition and diversity are influenced by host genotype and geography, with genetically similar individuals harboring more similar microorganisms, even within the same species [28]. But, individuals of bell sponges (*Ircinia campana*) show site-specific microbial profiles despite the lack of genetic differentiation of separate populations, suggesting that geography alone shapes microbial structure [28]. These findings point to multiple mechanisms of host influence, including vertical transmission of symbionts and functional trait variation, that govern the assembly of microbial communities and emphasize the importance of both interspecific and intraspecific host variation, as well as spatial heterogeneity, in mediating eco-evolutionary dynamics of host-associated microbial communities. Moreover, they also underscore the importance of systematically investigating how host traits and environmental gradients interact to shape microbial communities across hosts.

Mollusca is the second-largest animal phylum, with over 88 000 described species [32] that occupy an extraordinary wide range of habitats, from deep-sea hydrothermal vents and estuaries to freshwater lakes and terrestrial forests [32–34]. Molluscs possess diverse anatomical structures and life histories, making them excellent models for examining microbiome–host interactions. Their microbiomes have been implicated in critical functions such as chemosynthesis [35, 36], bioluminescence [37], pathogen defense [38], and nutrient assimilation [39].

Classic examples include the symbiosis between the bobtail squid (*Euprymna scolopes*) and a Gram-negative bacterium (*Vibrio fischeri*) [37], and the chemosynthetic associations of vent-dwelling gastropods and bivalves [40–42]. These examples further illustrate the ecological and functional importance of mollusk–microbe associations. In deep-sea hydrothermal vent ecosystems, scaly-foot gastropods (*Chrysomallon squamiferum*) harbor endosymbiotic chemoautotrophic γ-proteobacteria in their esophageal gland, which provide the host with nutritional support via sulfur oxidation [40]. In the lucinid clam–sulfur-oxidizing bacteria symbiosis (Lucinidae), intracellular bacteria housed in gill bacteriocytes enable the host to thrive in sulfide-rich sediments through chemosynthesis [35]. These bivalves maintain facultative relationships with chemoautotrophic bacteria that vary in abundance depending on habitat and food availability, highlighting the plasticity of these symbioses. In addition, the naval shipworm (*Teredo navalis)* hosts cellulolytic γ-proteobacteria in its gills, which produce enzymes that digest lignocellulose-rich wood, an unusual dietary niche for a mollusk [35, 36, 43, 44]. Finally, the symbiosis that occurs in the dorid nudibranch (*Rostanga alisae*), in which bacteria of the genera *Labrenzia, Maritalea, Bradyrhizobium, Burkholderia, Achromobacter*, and *Stenotrophomonas* inhabit host epithelial cells. These symbionts provide nitrogen through fixation processes that support host survival and complement their nutrient-limited conditions of exclusive sponge prey (*Ophlytaspongia pennata*) [45]. These examples demonstrate the evolutionary depth and functional diversity of molluscan microbial partnerships and underscore the importance of symbiosis in molluscan adaptation to extreme and resource-limited environments.

Many functions are associated with mollusk−microbe interactions, but the factors that drive their microbiome composition are not well known. To address this knowledge gap, we performed a systematic review of the literature evaluating the microbial communities of mollusks. Our main goal was to synthesize what is known about the assembly of molluscan microbiomes and determine general patterns concerning the drivers of microbial structure to better understand what influences the microbial communities of mollusks. Finally, we identified knowledge gaps concerning the microbiomes of mollusks, determined taxonomic and methodological biases, and proposed future directions to fill these gaps.

**Figure 1:**
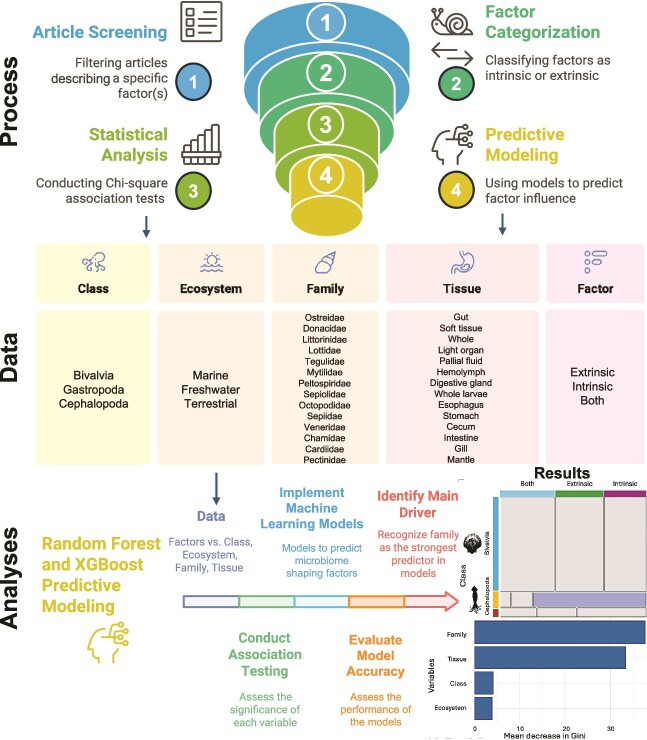
Flow diagram summarizing identification, screening, and eligibility assessment of articles; the data we extracted for each article; and the analyses conducted with the data (Supplementary Table S1).

We conducted a systematic literature search following Preferred Reporting Items for Systematic Reviews and Meta-Analyses (PRISMA)-style guidelines to identify peer-reviewed articles investigating factors shaping microbial community structure in mollusks (Fig. 1). Searches were performed in Google Scholar, PubMed, and Web of Science using the Boolean query: (*mollusk* OR *mollusc* OR *mollusca*) AND (*microbiome* OR *microbiota* OR *16S* OR *metabarcoding* OR *microbial community*) AND (*factor*) (literature reviewed until February 2025). The search yielded 1240 records, which were screened for duplicates, leaving 1010 unique entries. Titles and abstracts were evaluated for relevance to molluscan microbiomes, reducing the dataset to 120 studies for full-text assessment. Articles were included if they (i) examined microbiomes associated with mollusks and (ii) explicitly tested, identified, or inferred at least one intrinsic or extrinsic factor influencing microbial structure. Studies were excluded if they lacked factor attribution or did not characterize host-associated microbial communities. Because the reviewed studies employed diverse methodological approaches, including amplicon sequencing targeting different marker genes, variable sequencing platforms, and distinct analytical pipelines, methodological heterogeneity represents an inherent component of the current literature and may contribute to variation in reported microbiome patterns. A total of 85 studies met all criteria and were included in the final synthesis. We categorized drivers as host-associated traits (i.e. intrinsic factors such as host phylogeny, sex, tissue type) or environmental factors (i.e. extrinsic factors such as geography, depth, diet, temperature) to determine their importance in the assembly and maintenance of mollusk microbiomes. For each study, we extracted host taxonomy, geographic location, tissue type(s), environments (i.e. marine, freshwater, or terrestrial), as well as the factor(s) to which they attribute the microbial community structure (i.e. intrinsic or extrinsic) (Supplementary Table S1).

For the meta-analysis, we then performed association testing (chi-square, Fisher’s exact test) to assess relationships between variables (e.g. taxonomy, environment, tissue type) and the factor(s) driving the microbial community structure. Finally, we used classification models with predictive modeling approaches (Random Forest and XGBoost modeling [46, 47]) to test whether we can predict if a host-related or environmental factor is shaping the microbiomes of molluscan hosts (Fig. 1, Meta-analysis and predictive modeling methods described below under the section General Pattern or Context-dependent Mollusk Microbiome).

### Microbiomes in mollusks: an overview

**Figure 2:**
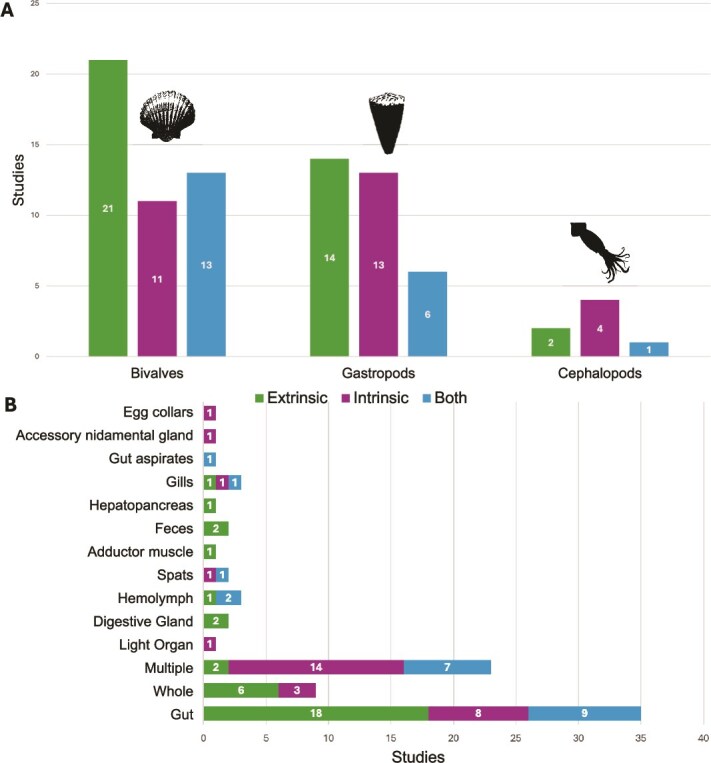
Total number of studies identifying factors per (A) Mollusca class (Bivalvia, Gastropoda, Cephalopoda) and per (B) tissue type, classified in studies that define host-related (intrinsic), environmental (extrinsic), or both factors as drivers of differences in microbial communities.

We identified and analyzed 85 studies that explicitly determined how host-related traits or environmental factors influence the microbial composition of mollusks. We only found studies for three of the eight Mollusca classes—Bivalvia (*n* = 45), Gastropoda (*n* = 33), and Cephalopoda (*n* = 7) (Fig. 2) and did not find any for Solenogastres, Caudofoveata, Monoplacophora, Polyplacophora, and Scaphopoda. We observed a large disparity in terms of the number of studies for particular mollusk taxa, tissue types, and the geographic areas where the samples for the studies were collected.

**Figure 3:**
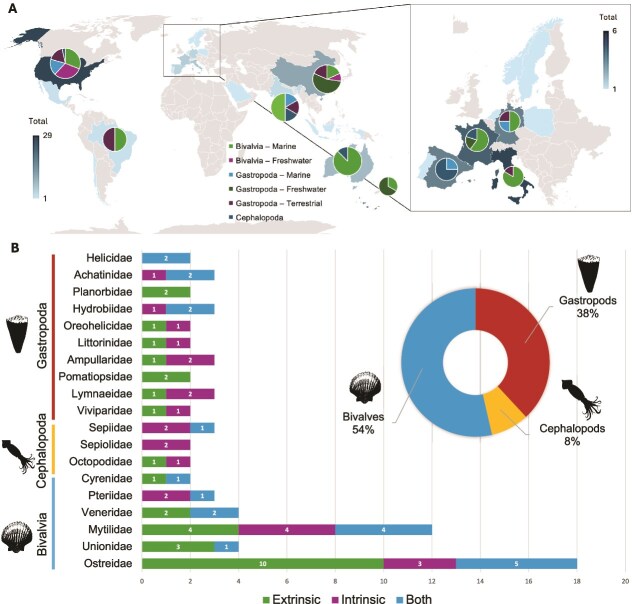
Geographic distribution and taxonomic scope of molluscan microbial community research across studies included in this review. (A) world map showing the geographic realm of studies on marine, freshwater, and terrestrial mollusks, with pie charts indicating the proportion of studies per molluscan class and ecosystem type at each location; map shading reflects the total number of studies per country (scale bar: 1–29). (B) Number of studies per molluscan family, grouped by class (Gastropoda, Cephalopoda, Bivalvia), showing whether intrinsic, extrinsic, or both factor types were identified as drivers of microbial community structure. The donut chart shows the proportion of total studies represented by each class (Bivalvia 54%, Gastropoda 38%, Cephalopoda 8%).

We expected to find the largest number of studies for gastropods, given that Gastropoda is by far the largest class of mollusks (~75 200 species) [32]. On the contrary, we found more studies for Bivalvia, the second largest class of mollusks (nearly 9900 species) [32]. This imbalance does not reflect true species richness but rather research priorities shaped by human use. Bivalves, including oysters, mussels, scallops, and clams, are commercially important, widely cultured, and frequently monitored for food safety and aquaculture performance. Consequently, they have become systems for host–microbe research, whereas the far more diverse gastropod lineages remain comparatively understudied. The studies of bivalves, though, were considerably taxonomically biased, coming from just six families and largely from two families with important aquaculture species (Fig. 3): Cyrenidae (*n* = 2), Pteriidae (*n* = 3), Veneridae (*n* = 4), Unionidae (4), Mytilidae (*n* = 12), and Ostreidae (*n* = 18). The studies we identified for gastropods (*n* = 33) do not appear biased toward any particular family, and include investigation of members of 17 gastropod families: Helicidae (*n* = 2), Achatinidae (*n* = 3), Planorbidae (*n* = 2), Hydrobiidae (*n* = 3), Oreohelicidae (*n* = 2), Littorinidae (*n* = 2), Ampullaridae (*n* = 3), Pomatiopsidae (*n* = 2), Lymnaeidae (*n* = 3), Limacidae (*n* = 1), Peltospiridae (*n* = 1), Trochidae (*n* = 1), Planorbidae (*n* = 3), Nassariidae (*n* = 1), Achatinellidae (*n* = 1), Ariolimacidae (*n* = 1), and Viviparidae (*n* = 2). For Cephalopoda (around 860 species [48]), we found seven studies across four families: Sepiidae (*n* = 3), Sepiolidae (*n* = 2), Loliginidae, and Octopodidae (*n* = 2).

Some studies included multiple tissues, or performed comparisons of microbial communities between tissues (*n* = 23), but most only analyzed a single tissue (*n* = 62, Fig. 2). The tissue type most commonly studied was the gut (*n* = 35), then the whole animal (*n* = 9), and other tissues with only one study each (i.e. pallial fluid, hemolymph, soft tissue, digestive gland, and light organ) (Fig. 2). In terms of environment, most studies (*n* = 53) focused on marine mollusks, and a few others focused on freshwater (*n* = 20) and terrestrial (*n* = 12) taxa. Most of these studies also largely involved mollusks from northern hemisphere countries, including the USA (*n* = 29), China (*n* = 11), Italy (*n* = 6), France (*n* = 5), Spain (*n* = 4), and Germany (*n* = 4) (Fig. 3). Studies of mollusks of Latin America (*n* = 5), Eastern Europe (*n* = 0), and Africa (*n* = 0) are less common. For factors shaping microbiomes of mollusks, studies identified intrinsic factors (*n* = 29), extrinsic factors (*n* = 35), or both factor types (*n* = 20) as the main drivers of microbial structure (Figs 2 and 3).

#### Bivalves

Among mollusks, bivalves are the most extensively studied group for microbiome research (Figs 1 and 2). The class includes economically and ecologically significant taxa such as mussels, oysters, clams, and scallops—organisms that are heavily harvested worldwide. We identified studies that characterize bivalve microbial communities, with many explicitly examining either intrinsic (*n* = 11) or extrinsic (*n* = 21) factors as drivers of microbial structure (Fig. 2).

##### Influence of extrinsic factors

Environmental variables such as microhabitat, temperature, season, and temporal dynamics were frequently reported as major determinants of microbiome composition of bivalves. For example, geographic location exerts a stronger influence than host species identity [49, 50]. Microbial communities of mussels vary more by habitat than by species [49], whereas in oysters, microhabitat is key [50]. Wild and farmed bivalves also exhibit differences in microbiome composition and diversity, in which farmed individuals have lower microbial diversity than wild conspecifics, and origin (i.e. wild vs. farmed) is a key determinant of microbial community structure [51, 52]. In contrast, microbial communities of digestive gland and gut of wild and hatchery-reared mussels (*Villosa nebulosa* and *Mytilus chilensis*, respectively) exhibit higher diversity, with community composition influenced mostly by geography, habitat, and cultivation method [51].

Temperature has been frequently linked to variation of microbes of bivalves. For example, although temperature does not significantly alter phylum-level microbiome composition of Korean mussels (*M. coruscus)*, it does affect relative abundances at the genus level [53]. Moreover, long-term shifts in microorganism composition of the Gould beanclam (*Donax gouldii)* occurred over a decade of rising temperature and salinity, whereas microbial diversity remained stable, thus indicating a dynamic but resilient community [54]. Seasonal water temperature changes are associated with fluctuations in microbial richness and community structure of oysters (*Crassostrea virginica*), in which additional taxa increase the diversity of core gut and pallial fluid microbiomes during warmer months [55].

Only a few studies evaluated the temporal dynamics of microbiomes of bivalves. Two showed strong seasonal effects, although another showed no effect. For example, microbial communities of the eastern oyster (*C. virginica*) exhibit lower levels of alpha diversity during the winter [55]. The microbiome structure of freshwater mussels (Unionidae) changes temporally and with host development [56]. Nonetheless, although the microorganisms of *D. gouldii* show long-term turnover over the course of a decade, their diversity remains stable over the short term despite seasonal environmental fluctuations [54].

In bivalves, shifts in microorganisms associated with extrinsic factors are reflected in changes in relative abundances, taxonomic turnover, and overall phylogenetic composition of the microbial communities. Comparisons between natural environments and non-natural conditions, including laboratory settings, aquaculture facilities, and hatchery-reared versus wild individuals, reveal changes in taxa such as *Tenericutes* (particularly class *Mollicutes*), *Mycoplasma*, *Psychrilyobacter*, *Vibrio*, *Fusobacteriaceae*, *Spirochaetes*, *Arcobacter*, and α-*Proteobacteria* [50–52]. Environmental variables such as temperature, collection site, and season further structure bivalve microorganisms, with shifts observed in *Bacteroides*, *Arcobacter*, *Helicobacteraceae* (including *Blastopirellula* and *Rubripirellula*), *Gammaproteobacteria*, *Bacteroidetes*, *Vibrio*, and *Pseudomonas*. These microbiome changes are frequently linked to environmental stress, seasonal variation in filtration and pumping activity, and, in some cases, increased susceptibility to secondary infections or proliferation of opportunistic bacteria, ultimately influencing host health and mortality [53–55].

##### Influence of intrinsic factors

We observed more frequent reporting of extrinsic (*n* = 21) than intrinsic (*n* = 11) factors as microbial community drivers in bivalves. Nonetheless, host-related traits, particularly host phylogeny, also emerged as an important predictor in which bivalve phylogenies are mirrored by similarity in their gut microbiome (i.e. show correlations between host phylogeny and microbiome structure, a phylosymbiotic pattern). Thick-shelled river mussels (*Unio crassus*) exhibit a strong phylogeographic pattern in microbial composition and a species-specific core microbiota, which shows that both environmental and host-specific factors shape microbiomes [57]. In addition, eastern oysters (*C. gigas*) and Mediterranean mussels (*M. galloprovincialis*) appear to have coevolved with members of their microbiomes [58]. Life stage is another intrinsic factor shaping microbial communities. In particular, microbial diversity declines as oysters (*C. corteziensis, C. gigas, C. sikamea*) progress from larval to adult stages [59] which reinforces the key role of ontogeny in structuring the microbial communities of bivalves [60, 61].

When life stage-specific shifts in microbial communities were described, postlarval stages often harbored higher microbial diversity and richness than adults of the same species. These ontogenetic differences are reflected in changes in the relative abundance of bacterial groups, including *Proteobacteria* (*Burkholderia* and *Escherichia*); *Bacteroidetes*; *Actinobacteria* (*Propionibacterium*); and marine-associated genera such as *Neptuniibacter*, *Marinicella*, *Rhodovulum*, and *Oceanicola*. In addition, genera such as *Vibrio* and *Pseudomonas* vary across life stages, with different life stages having different relative abundance of these genera [59, 60]. Beyond ontogeny, when bivalves exhibit microbiome host specificity, taxa such as *Vibrio* spp. play contrasting roles across bivalve hosts [58]. These microorganisms are described to contribute to host health in some species and be associated with disease in others, highlighting the context-dependent nature of host–microbe interactions in bivalves.

#### Gastropods

Gastropoda, the largest class within the phylum Mollusca, comprises >75 200 species and over 85.2% of extant mollusks [48, 62]. This group includes a diversity of body types that occupy marine, freshwater, and terrestrial environments. Despite their ecological and taxonomic breadth, only 17 families of gastropods have been the focus of studies describing factors driving differences in their microorganisms (Fig. 3). We identified 33 studies, divided between those investigating extrinsic (*n* = 14) and intrinsic (*n* = 13) factors shaping gastropod-associated microbiota (Fig. 2).

##### Influence of extrinsic factors

Environmental factors such as geography and habitat appear to be dominant influences on microbial community structures of gastropods. In multiple species of intertidal gastropods (Littorinidae), geographic location had a stronger effect on microbiome composition than interspecific differences at the same site [63]. In addition, geographic location explained more variation in gut microbiome composition of Rocky Mountain snails (*Oreohelix strigosa*) than collection year, highlighting the long-term stability of geography-associated microbial signatures. Site-specific environmental factors, such as drought index and habitat conditions, were also predictors of microbial variation. These findings support the hypothesis of horizontal transmission of microbial communities of terrestrial snails, in which they acquire their microbiota from their surrounding environment.

Microbiomes in gastropods affected by extrinsic factors such as geography exhibited changes in specific operational taxonomic units (OTUs) of phyla such as *Tenericutes* (*Mollicutes*), *Actinobacteria*, *Cyanobacteria*, *Clostridia* (*Firmicutes*), and *Planctomycetes*, and OTUs of family *Enterobacteriaceae*, and species *Sphingobacterium faecium*, *Pseudomonas* sp., and *Delftia* sp. vary over time [63, 64]. The distinctiveness of microbial communities across different habitats further supports the idea that local environmental conditions play a central role in shaping gastropod microbial communities [64, 65].

##### Influence of intrinsic factors

Host-related factors such as tissue type, host species, age, reproductive strategy (sexual vs. asexual), and life or developmental stage also drive microbiome composition. For example, obligately asexual and obligately sexual populations of New Zealand freshwater mud snails (*Potamopyrgus antipodarum*) harbor distinct microbial communities [21], which suggests that host reproductive mode influences microbiome structure, possibly through effects on host physiology or behavior. Although geography is a strong determinant of gut microbiomes of *O. strigosa*, as discussed above, they also differ significantly among embryos and adults [66].

Several gastropods from freshwater, terrestrial, and marine environments possess organ-specific microorganisms. For example, various tissues, including stomach, ovotestis, hepatopancreas, and gut, of two species of freshwater ram’s horn snails (*Biomphalaria alexandrina* and *B. glabrata*) show differences in their microbial communities [67]. Apple snails (*Pomacea canaliculata*) exhibit distinct microorganisms across different regions of their gut [68]. The deep-sea bone-eating snail (*Rubyspira osteovora*), apart from having a microbiome different from its environment and other deep-sea animals, has tissue type as a significant predictor of microbial disparity [69]. Beyond tissue-level variation, host species affects the structure of gastropod microbial communities. For example, three species of freshwater snails from Northeast Asia (*Sinotaia quadrata*, *Boreoelona ussuriensis*, *Radix plicatula*) that share habitat and feeding habits harbor distinct gut microbiomes despite overlapping diets and environment [70]. Microbes vary with intrinsic host factors operating at multiple biological taxonomic levels. When gastropods exhibit tissue-specific or species-specific microbiomes, the relative abundance of bacterial families such as *Aeromonadaceae*, *Mollicutes*, *Porphyromonadaceae*, and *Leptotrichiaceae*, as well as genera including *Ochrobactrum*, *Sediminibacterium*, *Lelliottia*, *Romboutsia*, and *Clostridium,* varies across tissues and species. Distinct microbial communities among gut regions (e.g. stomach, buccal mass, intestine) and between host species indicate niche partitioning within hosts and suggest that these microorganisms perform specialized functions tailored to particular tissues and host lineages [68, 70].

In contrast, differences in reproductive mode (sexual versus asexual) are associated with shifts in key bacterial groups such as *Rickettsiales* and *Rhodobacterales*, contributing to microbiome structure. Likewise, comparisons between fetal and adult life stages reveal marked changes in the relative abundances of *Verrucomicrobia*. The presence or absence of specific taxa across developmental stages suggests a combination of horizontal acquisition from the environment and vertical transmission in taxa present in all life stages [21, 66]. Together, these findings illustrate that microbiome composition is structured at multiple biological scales, not only by tissue type within a host but also by host species across sympatric taxa.

#### Cephalopods

Cephalopoda comprises a relatively small but ecologically and evolutionarily distinctive group within Mollusca, including squids, octopuses, and cuttlefish. Despite their biological importance and presence of well-characterized host–microbe interactions (e.g. light-organ symbioses) [37], cephalopods remain comparatively underrepresented in microbiome studies. Compared to the previous two classes, microbial communities of cephalopods have been understudied in terms of describing specific factors or predictors and even microbial diversity. We only found seven studies explicitly describing host-related drivers (*n* = 4), environmental factors (*n* = 2), or both (*n* = 1) shaping the microbiome composition of cephalopods.

##### Influence of extrinsic factors

Environmental and dietary inputs shape cephalopod-associated microbiomes in some cases. The microbial composition of the gut of the whiparm octopus (*Octopus variabilis*) is influenced by sampling location and season, highlighting the role of geographic and temporal dynamics [71]. Likewise, the diversity and community evenness of the gut microbiota of wild and captive paralarvae of the common octopus (*O. vulgaris*) are strongly influenced by diet [24]. Contrasting wild and captive octopus microbiomes, families such as *Corynebacteriaceae*, *Rivulariaceae*, *Mycoplasmataceae*, and *Vibrionaceae* change across different life stages. Wild paralarvae harbored more diverse and even microbial communities, whereas captive individuals rapidly converged toward low-diversity assemblages dominated by *Mycoplasmataceae* and *Vibrionaceae*. These shifts were attributed primarily to aquaculture conditions, including restricted diets, reduced exposure to environmental microorganisms, and stable rearing environments that favor fast-growing opportunistic taxa. In contrast, bacterial families such as *Comamonadaceae*, abundant in wild paralarvae, were nearly absent in captive individuals, highlighting the importance of environmental acquisition and diet in structuring cephalopod-associated microbiomes [24].

When microbial communities in cephalopods vary with sampling season and geographic site, groups such as *Bacteroidetes* predominate in warmer months and *Proteobacteria* in cooler sampling periods. Moreover, the abundance of potential pathogens such as *Vibrio vulnificus* increases in higher temperatures, indicating that extrinsic drivers like temperature can favor opportunistic taxa [71]. These findings reinforce that environmental filtering and context-dependent extrinsic factors, such as season and temperature, can shape cephalopod microbial communities, with particular implications for host health.

##### Influence of intrinsic factors

Sex, tissue type, and host phylogenetic relationships are associated with differences in cephalopod microbial communities. For example, male and female octopuses (*O. vulgaris*) exhibit different skin and mantle-associated mucus microbiomes [72]. In addition, although microbiomes associated with the accessory nidamental gland of nine squid species (*Euprymna berryi*, *E. morsei*, *E. scolopes*, *E. tasmanica*, *Eumandya parva*, *Doryteuthis pealeii*, *Sepioteuthis lessoniana*, *Uroteuthis duvaucelii*, and *Idiosepius pygmaeus*) and two cuttlefish species (*Sepia escuelenta* and *S. officinalis*) are distinct, the microbial communities of the squid species exhibit a pattern of phylosymbiosis in which differences in microbial composition are correlated with phylogenetic distances [73]. Gut microbiomes of various cephalopods, including octopuses (*O. variabilis* and *O. vulgaris*), squids (*Loliolus beka*, *Todarodes pacificus*, *Uroteuthis edulis*), and cuttlefish (*S. esculenta*), also exhibit phylosymbiosis [74].

When sex-specific differences in microbiomes have been reported in cephalopods, female skin-associated microbiomes are dominated by *Firmicutes*, particularly members of the order *Mycoplasmatales* and the genus *Lactococcus*, whereas males harbor microorganisms largely composed of *Proteobacteria*, with *Rhizobiales* and *Rhodobacteriales* as dominant taxa. Such a pattern has been hypothesized from differences in hormone profiles, skin and mantle chemistry, behavior, or ecological exposure between females and males, which may create distinct microhabitats favoring different microbes [72]. Across multiple squid and cuttlefish species, bacterial groups like *Alphaproteobacteria*, *Gammaproteobacteria*, and *Flavobacteriia* dominate, but the relative abundance and presence of specific taxa such as *Opitutae* (*Verrucomicrobia*) and *Ruegeria* (*Alphaproteobacteria*) differ between cephalopod families [73]. These patterns reflect host evolutionary history rather than environmental filtering and highlight host-driven assembly of microbiomes in specialized tissues. The clustering of microbial communities by host evolutionary history supports a phylosymbiotic signal and indicates that deterministic host selection outweighs neutral processes, such as stochastic dispersal and drift, in shaping cephalopod symbiotic microbiomes.

### Multitaxon studies

We only found one study that directly compared microbiomes of marine and terrestrial hosts or compared among species of more than one mollusk class. In particular, a comparison of cephalopods with a marine bivalve (*C. virginica*), several marine gastropods (*Elysia chlorotica*, *R. osteovora*, *Bathymargarites* sp., and *Phymorhynchus* sp.), a land snail (*Achatinella mustelina*), and 62 marine fishes found that the environment has a stronger structuring effect on gut microbiomes than phylogeny because marine mollusks and marine fishes shared more similar gut microbiota than marine mollusks and terrestrial gastropods [74].

### Interplay between host traits and environment in structuring molluscan microbiomes

Although many of the studies reviewed here examined intrinsic and extrinsic factors independently, fewer assessed their interactive effects on molluscan microbiomes. Only a limited subset quantified the degree of interaction between these predictors (Figs 2 and 3), although available evidence suggests that both extrinsic and intrinsic factors jointly contribute to shaping microbial composition. Interactions between intrinsic and extrinsic factors were indicated as a potential driver of differences in the microbiomes of bivalves [75], cephalopods [74], and gastropods [76].

Oysters and mussels (*C. virginica* and *M. edulis*) exhibit species, seasonal, and temporal variation, with a core gut microbiome partially influenced by microorganisms of the environment (i.e. seawater). These results suggest a connection between external and intrinsic factors of bivalve microbiomes [75]. As described above, host phylogeny, habitat, and diet influence microbial community differences among cephalopods [74]. Similarly, host-related traits (host species) and extrinsic factors (collection sites) exhibit a mixed interaction for gastropods [76]. For example, two species of marine snails (*Juturnia kosteri* and *Pyrgulopsis roswellensis*) possess microorganisms that are shaped both by the collection site and the species of the host [76]. These examples highlight a significant interaction between host identity and environmental context in structuring microbial communities.

Across gastropods, bivalves, and cephalopods, microbial composition varies in response to both host and environmental factors. In all three groups, shifts in microbiome structure are frequently reflected as changes in the relative abundance of recurrent bacterial groups (Proteobacteria (e.g. *Vibrio*, *Pseudomonas*, *Rhodobacterales*), *Bacteroidetes*, *Firmicutes*, and *Mollicutes*) rather than complete taxonomic turnover. Gastropods show differentiation across tissues, life stages, and reproductive modes, with taxa such as *Rickettsiales*, *Verrucomicrobia*, *Aeromonadaceae*, and *Mollicutes* contributing to host- and organ-specific patterns. Bivalves, by contrast, are shaped by extrinsic factors linked to aquaculture, season, and temperature, with shifts involving *Vibrio*, *Arcobacter*, *Mycoplasmataceae*, and *Fusobacteriaceae*, often associated with stress, disease, or altered metabolic activity. Cephalopods exhibit taxa such as *Mycoplasmataceae*, *Vibrionaceae*, *Rhodobacteriales*, and *Rhizobiales* dominating under distinct environmental or physiological contexts. Together, these patterns indicate both shared microbial responders across Mollusca and class-specific microorganisms related to host biology and habitat.

Taken together, these findings underscore the complex and often context-dependent interplay between intrinsic and extrinsic factors in shaping mollusk microbial communities. Although many studies highlight either host-related or environmental influences in isolation, fewer have explicitly quantified their combined effects or interactions. This gap presents an opportunity to synthesize current knowledge and uncover broader patterns across taxa and ecosystems. To address this, we conducted a systematic analysis of published studies, aiming to empirically test associations between key biological and ecological variables and the type of factor, intrinsic or extrinsic, most strongly associated with microbiome structure.

### General pattern- or context-dependent mollusk microbiome

To complement our literature synthesis, we performed a quantitative meta-analysis of published studies of mollusk microbiomes (Fig. 1, Supplementary Tables S1 and S2). Specifically, we used association testing (chi-square and Fisher’s exact tests) to evaluate relationships between variables such as molluscan family, class, ecosystem, and tissue type, and whether microbial structure was attributed to intrinsic (host-related) or extrinsic (environmental) drivers across the included studies. In addition, we applied permutation-based feature importance analysis to identify which variables most strongly influence the classification of intrinsic versus extrinsic factors. Finally, we implemented supervised machine learning approaches, including Random Forest [77] and XGBoost classifiers [46, 78], to assess the predictive power of biological and ecological variables in determining the type of factor shaping the host-associated microbial communities. This integrative analysis provides new insights into generalizable patterns and potential biases in the current molluscan microbiome literature. However, because the dataset was constructed from published studies, the response variable represents how individual studies attributed microbial variation to intrinsic or extrinsic drivers rather than standardized effect sizes derived from raw microbiome data. Consequently, these quantitative analyses evaluate patterns in reported drivers across studies rather than directly measuring microbial composition variation.

#### Association testing

Among all tested variables, host family showed the strongest association across studies with microbiome composition (Pearson’s chi-square test, *P-*value = 1.43 × 10^−5^, X-squared = 138.9) and then host class (Pearson’s chi-square test, *P-*value = .004, X-squared = 14.9). In contrast, variables such as tissue type (Pearson’s chi-square test, *P*-value = .054, X-squared = 112.5) and ecosystem (Pearson’s chi-square test, *P*-value = .499, X-squared = 3.4) showed weaker and less significant associations. Tissue types (e.g. gill vs. digestive gland) were associated with significant shifts in microbial composition in certain taxa, but these effects were less generalizable across studies. These results suggest that among the factors examined, host taxonomy exerts the strongest and most reproducible influence on the structure of microbial communities in mollusks.

#### Predictive modeling

**Figure 4:**
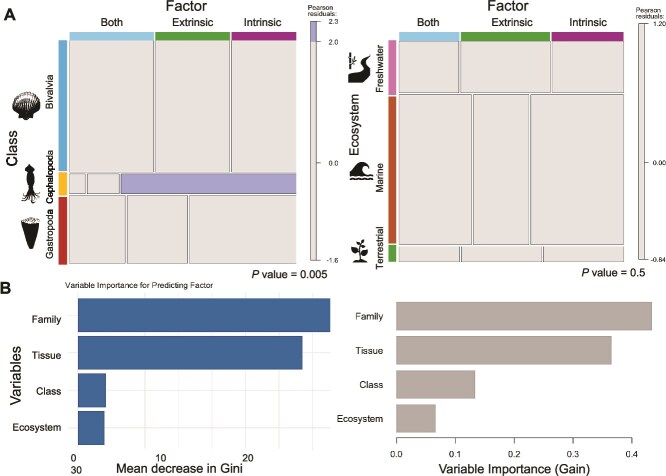
Pattern of association between host variables and reported microbial community drivers across molluscan studies. (A) Association mosaic plots for class (left) and ecosystem variables (right). (B) Variable importance plot for predicting factors with random forest (left) and XGBoost model prediction (right). Mean decrease in Gini values: higher values indicate greater importance of the variable (family, tissue, class, ecosystem) in classifying the factor (intrinsic or extrinsic).

To further quantify the strength of host-associated microbial signatures and evaluate their potential as diagnostic markers, we implemented supervised machine-learning models to determine if, based on previous variables (e.g. taxon, ecosystem, tissue type), we can predict whether a host-related (intrinsic) or environmental (extrinsic) factor is shaping the microbiomes of the molluscan host. We encoded the primary driver identified by each study (*n* = 85) as a binary response variable (“intrinsic” vs. “extrinsic”). Predictor variables included host class, family, tissue type, and ecosystem (Fig. 1, Data, Supplementary Table S1). Studies that included more than one molluscan class, family, or tissue type were recorded multiple times in our dataset to capture variation across these categories (Supplementary Table S1). Because multiple entries sometimes originate from the same study, such observations are not fully independent and therefore increase the representation of certain studies in the dataset. Consequently, the patterns identified here should be interpreted as exploratory and descriptive of trends reported across the literature rather than as fully independent statistical estimates. Models were trained using 10-fold cross-validation repeated five times to minimize sampling bias. Overall statistics and statistics by class are reported in Supplementary Table S2. A Random Forest classifier achieved moderate classification accuracy (*P*-value = .021, 60% (CI = 40.6%–77.3%), indicating the model performed better than random guessing with cross-validated models correctly classifying samples into their respective host classes with consistent performance metrics (e.g. precision, recall, and F1 scores across all categories; Fig. 4B). These results were robust across different random seeds and balanced training datasets, indicating that models did not overfit to study-specific artifacts. Similar performance was obtained using XGBoost (*P*-value = .00085, overall accuracy = 0.7, 95% CI [0.506, 0.8527]), a gradient-boosted tree method optimized for classification (Fig. 4B). In both models, host family showed the highest mean decrease Gini score, indicating that this variable contributed most strongly to classification among the predictors considered across studies in distinguishing environmental and/or host-related factors potentially reflecting ecological specialization or host filtering.

### Biases, patterns, and gaps in mollusk microbiome research

Our synthesis reveals substantial disparities in the scope and focus of molluscan microbiome research. Most studies have focused disproportionately on bivalves, particularly species used in aquaculture. This taxonomic bias, alongside limited geographic, temporal, and tissue-based diversity, constrains our understanding of the broader evolutionary and ecological patterns that shape molluscan microbiomes. Since 2006, studies on bivalve-associated microbial communities have increased exponentially, with many identifying significant influences of both intrinsic (e.g. host phylogeny, tissue type) and extrinsic (e.g. geography, environment) factors (Figs 2 and 3). Several studies demonstrate strong host-specific microbial associations, especially in cephalopods and bivalves, hinting at potential species-level co-evolution and host filtering of microbial partners (e.g. bobtail squid-*Vibrio* [37]).

A further consequence of these biases is that the apparent predominance of intrinsic drivers may, in part, reflect uneven sampling across molluscan groups and ecological contexts. Studies on aquaculture-associated bivalves dominate the current literature and are frequently studied under controlled or semi-controlled conditions, where host identity, developmental stage, and tissue type are contrasted. Such designs may enhance the detection of host-specific microbial communities and therefore amplify signals consistent with deterministic host filtering. In contrast, wild mollusk taxa, particularly gastropods and cephalopods, remain comparatively undersampled, and studies in natural conditions often encompass greater environmental heterogeneity that may alter the relative importance of host versus environmental effects. As a result, the influence of intrinsic factors identified here should be interpreted within the context of existing taxonomic and ecological biases, highlighting the need for broader sampling across wild populations, habitats, and underrepresented lineages to assess microbiome assembly more accurately across Mollusca.

Our meta-analysis confirms that host traits, especially phylogeny at the family and class levels, are the most consistent predictors of microbial composition across mollusks. Statistical association testing highlights host family as the factor most strongly linked to whether studies identify intrinsic drivers, whereas extrinsic factors such as geography and methodology showed more variable and context-dependent effects. Predictive modeling further supports this pattern, showing moderate classification performance, suggesting that machine learning models reliably classified host taxa based on microbiome data. The identification of host family and tissue type as the variables most strongly associated with reported microbial community drivers is also consistent with the concept of phylosymbiosis [12], in which microbial similarity reflects host evolutionary relationships. Although our analyses do not directly measure phylogenetic congruence between hosts and microbiomes, the prominence of host taxonomy across studies suggests that eco-evolutionary processes such as host filtering may contribute to structuring molluscan microbial communities. Yet, although host filtering appears prominent in some bivalves and cephalopods, the relatively sparse data on gastropods and other molluscan lineages limit our ability to generalize these patterns across the phylum. Together, these results highlight consistent patterns in how molluscan microbiome drivers are reported across the literature while also underscoring the need for standardized comparative datasets that directly quantify microbial composition across taxa, tissues, and environments.

A recent review of molluscan microbiomes similarly underscores the ecological importance of these microbiomes and highlights dominant taxa such as *Proteobacteria*, *Bacteroidetes*, and *Firmicutes* across gastropods, bivalves, and cephalopods [79]. Although they frame these broad patterns in terms of functional roles, our results extend this understanding by explicitly identifying intrinsic and extrinsic drivers that generate these community patterns and by quantifying their relative contributions using statistical and predictive modeling. By linking taxonomic shifts not only to host function but also to ecological assembly processes (deterministic environmental filtering, stochastic dispersal, and host phylogeny), we provide a complementary perspective that helps explain why such functional patterns emerge. This integration of taxonomic, functional, and ecological drivers is critical for improving predictions of microbiome responses under environmental change and for advancing their application in aquaculture, conservation, and environmental monitoring.

Understanding the interplay between intrinsic and extrinsic drivers is especially urgent in the context of global change. Accelerated climate shifts will likely disrupt host–microbiome interactions across taxa [26]. Increased anthropogenic and natural pressures, such as rising ocean temperatures, pollution, and acidification, may restructure microbial communities with unknown consequences for host health, physiology, and evolution. Environmental changes may further promote microbiome dysbiosis following the “Anna Karenina principle,” whereby stressed hosts exhibit greater interindividual variability in microbial composition as stable host–microbe associations break down [80]. Evidence from marine organisms indicates that microbiome resilience depends on both host condition and environmental stability, with stress often leading to increased microorganism variability, proliferation of opportunistic taxa, and reduced functional stability of host-associated microbiomes. Studies of corals and other marine invertebrates demonstrate that environmental disturbance can shift microbiomes from stable, host-regulated states toward more stochastic or stress-associated microbiomes, highlighting the importance of considering microbiome resilience and dysbiosis when predicting molluscan responses to ongoing environmental change [81, 82]. Improved baseline knowledge of host–microbiome dynamics will enhance our capacity to predict and mitigate these impacts.

This urgency is amplified by both sampling and methodological limitations identified in this literature survey. Most studies restrict sampling to a single tissue, typically the gut, but relatively few examine multiple tissues or whole-animal microbiomes, limiting our ability to distinguish tissue-specific from host-level patterns. In addition, methodological heterogeneity across studies, including differences in sequencing, taxonomic resolution, and analytical pipelines, can influence the detection and interpretation of microbial diversity. Most studies rely on bacterial 16S ribosomal RNA (rRNA) gene sequencing, with comparatively limited characterization of fungal or viral communities. Consequently, throughout this review, the term microbiome or microbial communities largely reflects bacterial assemblages, as other microbes such as fungi, viruses, and protists remain comparatively underexplored or underreported in molluscan systems. Broader anatomical sampling and integration of multi-omics approaches, including metagenomics, metatranscriptomics, and multi-domain surveys, will be essential for understanding ecological and functional drivers of molluscan microbiome assembly and to capture the full complexity of molluscan host–microbe associations.

An additional conceptual gap in current molluscan microbiome research is the limited application of neutral ecological models. Although many studies interpret microbial variation through deterministic forces (host filtering or environmental drivers), few evaluate whether observed patterns could instead emerge from stochastic dispersal and drift. The Sloan neutral model [9], in particular, has been used in other host–microbe systems for distinguishing neutral from non-neutral taxa and quantifying the balance between stochastic and deterministic assembly processes [83–85]. Applying such models to molluscan datasets would help clarify whether apparent species-specific or taxa-specific patterns arise from strong host selection or from neutral community dynamics shaped by habitat connectivity, larval dispersal, and environmental microorganisms.

## Conclusion

Our synthesis identifies a consistent yet nuanced pattern in the symbiotic associations of molluscan hosts: the composition of molluscan microbiomes is shaped by both intrinsic and extrinsic factors, with effects that are taxon- and context-dependent. Host phylogeny, particularly at the family and class levels, emerged as the most robust predictor of microbial composition, whereas environmental variables contributed to additional but often secondary variation. This finding underscores the need for a more integrative and taxonomically inclusive approach to microbial community research across Mollusca. To advance the field, we recommend a multi-factor strategy. First, efforts should be directed at an increased diversity of species, especially among underrepresented classes such as Polyplacophora, Scaphopoda, Monoplacophora, and Caudofoveata, with expanded geographic and temporal resolution. Second, studies should simultaneously evaluate both intrinsic and extrinsic factors, as their interaction likely governs community assembly. Third, we should leverage emerging sequencing and metagenomic tools to investigate functional relationships and co-evolutionary dynamics between hosts and symbionts. Fourth, future work should test neutral models of community assembly [8, 86], which have been largely absent from current analyses but may offer key insights into stochastic versus deterministic processes. Finally, we emphasize the importance of sampling beyond traditional tissues to understand how microbial communities vary within individuals and across organ systems. Integrating comparative approaches with predictive modeling provides a useful framework for linking observed patterns in the literature with broader eco-evolutionary processes, including phylosymbiosis and host-driven microbial selection. These steps will help generate a more complete and predictive framework for understanding the ecological and evolutionary dynamics of host–microbiome interactions in one of the most diverse animal phyla on Earth.

## Supplementary Material

Supplementary_Material_wrag092

## Data Availability

All data generated or analyzed during this study are included in this published article and its supplementary information files.
