# Supplementary material for: Taxonomy, tissue, and habitat influence mollusk microbial communities
Source: ISME J. 2026 Apr 22;20(1):wrag092. doi: 10.1093/ismejo/wrag092 (PMC13157833; doi:10.1093/ismejo/wrag092)
Supplement: Supplementary_Material_wrag092 [file supplementary_material_wrag092.zip › Supplementary_legends_wrag092.pdf]

Figure Legends

Figure 1. Flow diagram summarizing identification, screening, and eligibility assessment of articles, the data we extracted for each article, and the analyses conducted with the data (Supplementary Table 1).

Figure 2. Total number of studies identifying factors per **A** Mollusca class (Bivalvia, Gastropoda, Cephalopoda) and per **B** tissue type, classified in studies that define host-related (intrinsic), environmental (extrinsic), or both factors as drivers of differences in microbial communities.

Figure 3. Geographic distribution and taxonomic scope of molluscan microbial community research across studies included in this review. **A** World map showing the geographic realm of studies on marine, freshwater, and terrestrial molluscs, with pie charts indicating the proportion of studies per molluscan class and ecosystem type at each location; map shading reflects the total number of studies per country (scale bar: 1–29). **B** Number of studies per molluscan family, grouped by class (Gastropoda, Cephalopoda, Bivalvia), showing whether intrinsic, extrinsic, or both factor types were identified as drivers of microbial community structure. The donut chart shows the proportion of total studies represented by each class (Bivalvia 54%, Gastropoda 38%, Cephalopoda 8%).

Figure 4. Pattern of association **between host variables and reported microbial community drivers across molluscan studies. A Association** mosaic plots for class (left) and ecosystem variables (right). **B** Variable importance plot for predicting factors with Random Forest (left) and

XGBoost model prediction (right). Mean decrease in Gini values: higher values indicate greater importance of the variable (family, tissue, class, ecosystem) in classifying the factor (intrinsic or extrinsic).

Supplementary Table Legends

Supplementary Table S1.

Data extracted from each article included in the systematic review of factors shaping molluscan microbial community structure. For each study, recorded variables include host taxonomy, geographic location, tissue type, ecosystem, and the factor(s) attributed to microbial community structure (intrinsic, extrinsic, or both). Studies encompassing more than one molluscan class, family, or tissue type were recorded as multiple entries to capture variation across these categories.

Supplementary Table S2.

Classification performance statistics for Random Forest and XGBoost predictive models trained to distinguish intrinsic from extrinsic drivers of molluscan microbial community. Overall model statistics and performance metrics by molluscan class (Bivalvia, Gastropoda, Cephalopoda) are reported, including accuracy, precision, recall, and F1 scores.
